# Stereochemical Criteria for Prediction of the Effects of Proline Mutations on Protein Stability

**DOI:** 10.1371/journal.pcbi.0030241

**Published:** 2007-12-07

**Authors:** Kanika Bajaj, M. S Madhusudhan, Bharat V Adkar, Purbani Chakrabarti, C Ramakrishnan, Andrej Sali, Raghavan Varadarajan

**Affiliations:** 1 Molecular Biophysics Unit, Indian Institute of Science, Bangalore, India; 2 Department of Biopharmaceutical Sciences and Pharmaceutical Chemistry, University of California San Francisco, San Francisco, California, United States of America; 3 Chemical Biology Unit, Jawaharlal Nehru Center for Advanced Scientific Research, Jakkur, Bangalore, India; University of California San Diego, United States of America

## Abstract

When incorporated into a polypeptide chain, proline (Pro) differs from all other naturally occurring amino acid residues in two important respects. The φ dihedral angle of Pro is constrained to values close to −65° and Pro lacks an amide hydrogen. Consequently, mutations which result in introduction of Pro can significantly affect protein stability. In the present work, we describe a procedure to accurately predict the effect of Pro introduction on protein thermodynamic stability. Seventy-seven of the 97 non-Pro amino acid residues in the model protein, CcdB, were individually mutated to Pro, and the in vivo activity of each mutant was characterized. A decision tree to classify the mutation as perturbing or nonperturbing was created by correlating stereochemical properties of mutants to activity data. The stereochemical properties including main chain dihedral angle φ and main chain amide H-bonds (hydrogen bonds) were determined from 3D models of the mutant proteins built using MODELLER. We assessed the performance of the decision tree on a large dataset of 163 single-site Pro mutations of T4 lysozyme, 74 nsSNPs, and 52 other Pro substitutions from the literature. The overall accuracy of this algorithm was found to be 81% in the case of CcdB, 77% in the case of lysozyme, 76% in the case of nsSNPs, and 71% in the case of other Pro substitution data. The accuracy of Pro scanning mutagenesis for secondary structure assignment was also assessed and found to be at best 69%. Our prediction procedure will be useful in annotating uncharacterized nsSNPs of disease-associated proteins and for protein engineering and design.

## Author Summary

Unlike other amino acids that constitute proteins, Proline is missing a vital hydrogen atom and also bestows local structural rigidity to the three-dimensional (3D) structure of proteins. In some locations, proline can be introduced with little or no detrimental effect to protein function, while at others it is destabilizing and can result in significant degradation or aggregation of the protein. To determine the features of protein 3D structure that tolerate the introduction of prolines, each of the 101 amino acid residues of the protein CcdB were replaced with Proline, and the functional consequence of the mutations were observed. On correlating these data to features of protein 3D structure, a decision tree was generated to predict the functional consequences of proline mutations in proteins of known (or accurately modeled) 3D structure. The performance of the tree was assessed on three different datasets that contained a total of 289 proline mutants in 37 different proteins. The average accuracy of prediction was 75%. The decision tree will be useful in predicting if known but uncharacterized proline mutations in disease-related proteins are likely to have adverse effects. It will also be useful in engineering and designing new proteins and peptides.

## Introduction

Proline (Pro) is unique among the 20 naturally occurring amino acid residues. On the one hand, because Pro lacks an amide proton the main chain amide N is incapable of forming H-bonds (hydrogen bonds). Hence, substituting a residue involved in a main chain H-bond with Pro could destabilize the protein. This property has previously been exploited to obtain information about residues involved in secondary structure [1–3]. On the other hand, the rigid pyrrolidine ring constrains the main chain dihedral angle φ to a narrow range of values close to −65°. It has also been observed [4–6] that Pro restricts the conformation of the residue preceding it in a protein sequence. The Ramachandran map of the pre-proline residue has a large excluded area between −40° < ψ < 50°. This restricts the conformation of the α_L_ and α regions. There is also a small leg of density in the β region that is unique to pre-proline residues. Hence, Pro can potentially increase protein stability because it decreases the conformational entropy of the denatured state. In addition, Pro is usually conserved in proteins and often plays an important role in protein structure and function [5,7,8].

Previous studies on Pro mutants of different proteins have shown that the thermodynamic effects of introducing Pro depend on various factors including residue position (accessibility and secondary structure), φ value of the original residue, H-bonding of the amide group of the original residue, and electrostatic or hydrophobic interactions of the original residue [1,5,9–12]. However, it is not yet clear whether the introduction of Pro at a given position in a protein will have a perturbing (destabilizing) or nonperturbing effect on the thermodynamic stability of the protein. The aim of the present work is to generate an algorithm based on Pro scanning mutagenesis data which can be used to predict the perturbing/nonperturbing effect of Pro substitution at a given position for any globular protein. We also examine the utility of Pro scanning mutagenesis to infer protein secondary structure.

The experimental system used in this study, controller of cell division or death B protein (CcdB), is a 101 residue, homodimeric protein encoded by F plasmid. The protein does not contain any disulfides or metal ions. The protein is an inhibitor of DNA gyrase and is a potent cytotoxin in Escherichia coli (E. coli). Transformation of normal *E.coli* cells with plasmid expressing the wild-type (WT) CcdB gene results in cell death. If the protein is inactivated through mutation, cells transformed with the mutant genes will survive. In this work we attempted to replace each of 101 amino acids of homodimeric CcdB with Pro using high throughput mega-primer based site-directed mutagenesis. A total of 77 mutants could be generated. Mutant phenotype was assayed as a function of expression level by monitoring the presence or absence of cell growth as a function of inducer (arabinose) concentration. Based on an analysis of CcdB Pro scanning mutagenesis, phenotypic data, and its correlation with various structural parameters, a decision tree was created to classify Pro substitutions of a protein into perturbing (those which destabilize the protein) and nonperturbing (nondestabilizing) mutations. The decision tree was further validated on a large phenotypic dataset of 163 Pro mutants of T4 lysozyme at two different temperatures (37 °C and 25 °C), a nonsynonymous single nucleotide polymorphism (nsSNP) database of Pro substitutions which are associated with various diseases and on Pro substitutions extracted from the ProTherm database and literature.

## Results/Discussion

### Pro Scanning Mutagenesis of CcdB

**Figure 1:**
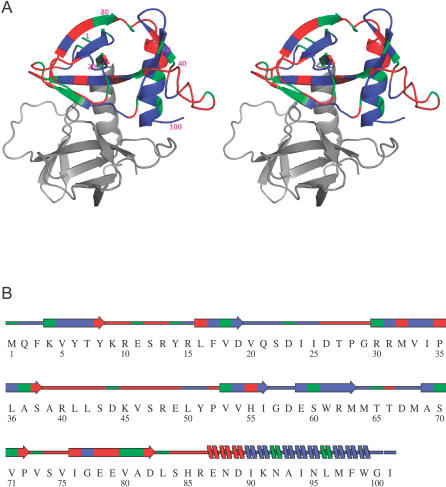
Location of Active and Inactive Pro Mutants of CcdB in the Context of the Overall 3D Structure of the Protein (A) In this stereo view, one of the protomers is shown in grey and the other in color. (B) Secondary structure representation of CcdB. Locations where Pro mutations could not be generated are shown in green, while red and blue represent locations of active and inactive mutants, respectively. Every 20th residue is labeled.

**Figure 2:**
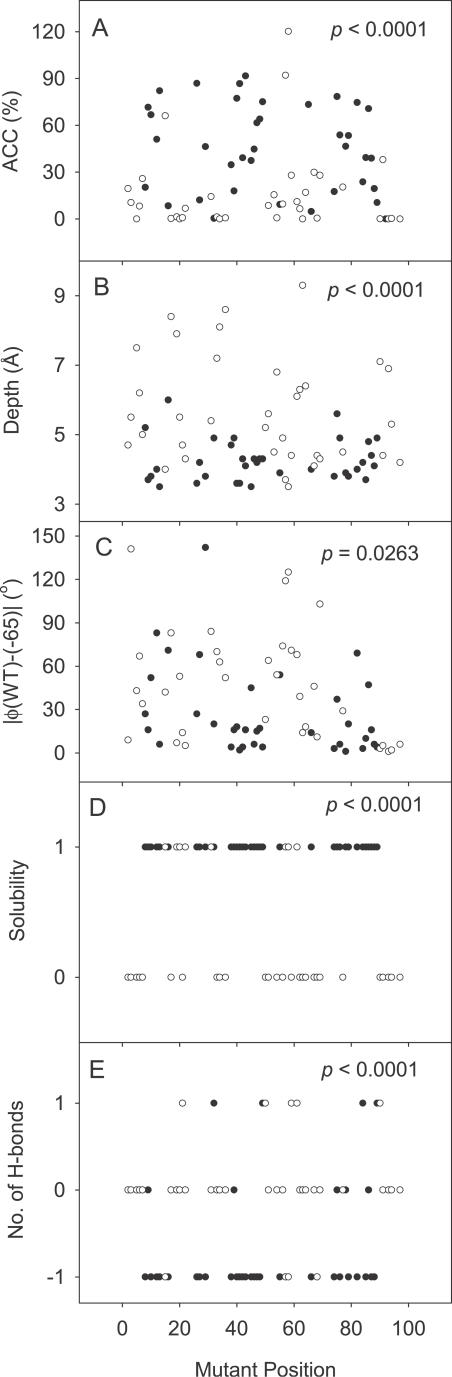
Correlation of Pro Mutant Activity with Various Structural Parameters of WT and Mutant Models of CcdB Solid circles represent active mutants and empty circles represent inactive mutants. (A) WT residue ACC, (B) WT residue depth, (C) |φ(WT) − (−65)| (D) solubility of mutant (0 denotes insoluble and 1 denotes soluble), (E) number of H-bonds formed by WT residue in native structure and acceptor in mutant models (−1 denotes WT amide not involved in H-bond, 0 denotes WT amide involved in H-bond but acceptor of this amide group not satisfied in any of the mutant models and 1 denotes acceptor forms new H-bond in at least one mutant model). Correlations with *p*-values less than 0.05 are considered statistically significant.

A total of 77 single site Pro mutants were generated out of the possible 97 (four of the 101 WT residues are Pro) positions of CcdB. Individual phenotypes for each mutant are shown in Figure 1 and Table S1. The phenotype of the Pro mutants was observed to be sensitive to expression level. At the lowest level of expression (0% arabinose), 45% of the mutants showed an active phenotype, while at the highest level of expression (0.1% arabinose), it increased to 74%. However, 50% and 80% of the mutants showed an active phenotype at the lowest and highest expression levels, respectively, if active site mutants were not considered. Table 1 summarizes the mutant phenotypes at low (0% arabinose) and high levels of expression (0.1% arabinose) along with their solubilities, examined as a function of ACC (percentage side chain solvent accessible surface area of a residue). We have previously shown that Ala and Asp scanning mutagenesis of CcdB can be used to identify active site residues [13]. At such sites, either the corresponding Ala and Asp mutants are inactive at both low and high inducer concentrations (residues 24, 98, 99, 100, and 101) or Ala is active but corresponding Asp is inactive and expression/solubility is unaffected (residues 25, 95). Analysis of the CcdB:DNA gyrase crystal structure [14] shows that residues 24, 25, 26, 87, 88, 91, 92, 95, 99, 100, and 101 are within 4 Å of DNA gyrase using the Structure Analysis module of CCP4 [15]. Thus, scanning mutagenesis data identifies a subset of these residues as being crucial for the CcdB:Gyrase interaction. Mutants belonging to this subset (residues 24, 25, 95, 98, 99, 100, and 101) were not considered for further analysis as Pro mutations at such active site residues can result in loss in activity without affecting stability. Sixteen residues at positions 2, 20, 21, 22, 25, 27, 32, 66, 68, 69, 94, 95, 97, 98, 99, and 100 are at the dimeric interface. Pro mutations at 12 of these 16 positions were inactive. These residues were not excluded from the analysis, as mutating dimerization interface residues can affect the stability of a protein and there is no good justification for treating dimerization interface residues differently from other buried residues. Of the ten mutants at buried positions but not at dimerization interface, all were inactive. Solubility data of Pro mutants (Table 1 and Figure 2D) was found to correlate with activity [13]. Seventy-seven percent (27 out of 35) of nonactive site mutants that showed an inactive phenotype at 0% arabinose were insoluble. Not surprisingly, the lowest fraction of active mutants were those with ACC < 5% and the highest fraction was for residues with ACC > 40% (Table 1).

**Table 1:**
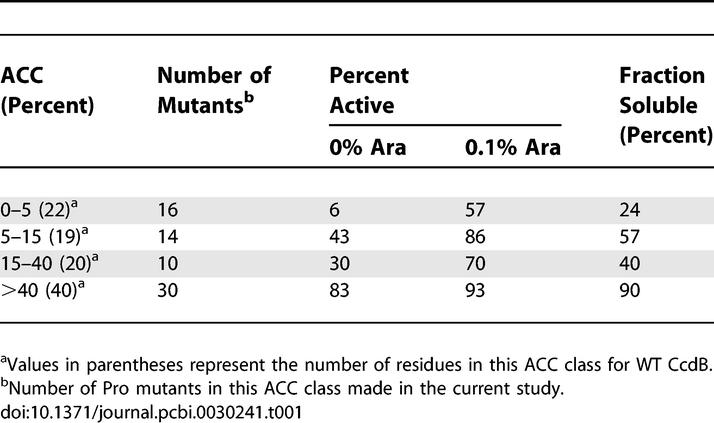
Fraction of Active and Soluble Mutants as a Function of Total Side Chain Accessibility (ACC) in the Absence and Presence of the Inducer Arabinose

### CcdB Secondary Structure Analysis

Pro mutants were divided into two classes, active (A) and inactive (I), depending on their phenotype at low and high expression levels. The correlations of Pro mutant activity with secondary structure and with involvement of the main chain amide of the WT residue in an H-bond were analyzed. Pro substitutions which show an active phenotype at both low and high expression levels are designated as nonperturbing (Class 1, Table 2). Those which show an inactive phenotype at low expression levels and either an active or an inactive phenotype at high expression levels are designated as perturbing (Class 2, Table 2). CcdB is a moderately stable protein (T_m_ = 61 °C, ΔG_u_° (298K) = 21 kcal/mol (1 cal ≈ 4.184 J) of dimer) [16]. It is assumed that the loss of activity upon mutating nonactive site residues implies that the mutant protein is thermodynamically less stable than the WT. This is supported by the observation that a large fraction of these mutants go into inclusion bodies when overexpressed. For stereochemical reasons, it is generally thought that Pro mutations are poorly tolerated in regions of secondary structure [5]. However, previous studies have demonstrated that Pro can be found at edge strands in non–H-bonded sites of antiparallel β sheets [17], and, indeed, aromatic-Pro interactions occur in sheets [18,19]. In addition, although Pro does not have the amide NH group, CH-O interactions can substitute for the normal H-bond to accommodate a Pro in the interior of the helix [20]. In case of CcdB, 12 of the 35 (34%) Pro mutations in regions of helix or β strand (as defined in the crystal structure—PDB [21] code 3vub [22])—are nonperturbing. Residues at the first three positions of helices typically do not have their amide protons involved in H-bonds. Even if these positions are ignored, nine of 32 Pro mutations in strands and helices are nonperturbing. Of these, two are the N-terminal residues and three are the C-terminal residues of strands. Pro mutations can therefore be nonperturbing even in regions of secondary structure. This is probably because Pro residues can be accommodated close to the ends of secondary structural regions where adjacent turns/loops can rearrange without high energetic cost. For example, Pro mutations at residues 8, 16, 38, 76, 82 (at either the ends or beginning of β strands) and residues 87, 88, 89 (at the N-terminus of an α helix) are all nonperturbing. Several H-bonded residues not in regions of secondary structure, e.g., residues 2, 3, 20, 21, 22, 25, 50, 51, 64, and 67 are intolerant to Pro substitution. Phenotypes of Pro mutants have previously been used to infer information about residues involved in secondary structure in proteins where no homology model or other structural information is available [1–3]. The present studies show that Pro scanning mutagenesis alone cannot be reliably used to obtain secondary structural information (Table 2 and Table S1). The accuracy of secondary structure assignment from Pro scanning mutagenesis was calculated in two different ways. In the first approach, it was assumed that at each of the 70 nonactive site residues, wherever substitution by Pro leads to loss of activity, the WT residue is in a region of secondary structure (helix or strand). Conversely, where Pro substitution is nonperturbing, the WT residue is in a region lacking secondary structure. The accuracy using this approach was 63% (Table S1). If secondary structure is assigned to regions by considering the average mutant phenotype in a three-residue window, the assignment accuracy is 69%. For example, if in a stretch of three nonactive site residues, two or more of the Pro substitutions are inactive, the middle residue is assigned to be in a region of secondary structure, else it is assumed to be in a region lacking secondary structure. These figures are lower than values of 75%–78% obtained from existing sequence-based computational methods of secondary structure predictions [23], although it should be noted that PSIPRED [24], a widely used secondary-structure prediction program only yielded a prediction accuracy of 42% when applied to CcdB. The figure of 69% described above masks the fact that the bounds of all secondary-structure elements are incorrectly assigned and one strand is missed out entirely. The accuracy of secondary-structure assignment is far lower than 69% if the accuracy measure were to combine measures of number of correctly predicted segments with correctness of predicted segments. It was recently shown [25] that Ala scanning combined with Pro scanning mutagenesis gives useful information about backbone conformation in amyloid fibrils. The Ala mutants were shown to be useful to identify cases where Pro mutations destabilized the fibril because of changes in side chain hydrophobicity rather than changes in the main chain backbone configuration. However, we find that for CcdB, Ala scanning mutagenesis results did not correlate with hydrophobicity changes as most Ala mutants at nonactive site positions showed an active phenotype [13].

**Table 2:**
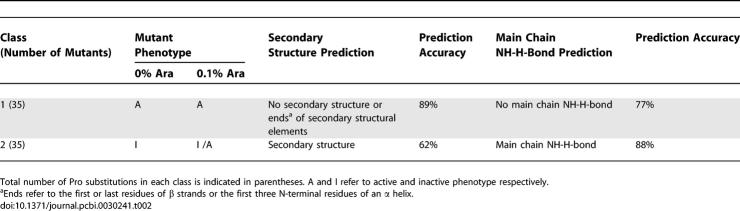
Secondary Structure and Main Chain H-Bond Prediction from Pro Scanning Mutagenesis

If the WT residue amide proton is involved in H-bonding, then substitution with Pro should lead to appreciable destabilization of the protein [26]. This is indeed the case (last column of Table 2). The data in Table 2 suggest that Pro scanning mutagenesis can provide information about a) a subset of residues that are not in regions of secondary structure or are at the ends of secondary structural elements, b) a subset of residues whose main chain amide protons form H-bonds. This information is useful in the absence of the 3D structure of a protein and can be used to discriminate between various model structures. However, Pro scanning mutagenesis has limitations when applied to precisely define regions of secondary structure as discussed above.

### Correlation between Pro Activity and Short Contacts

Assuming no main chain rearrangement, the number of short contacts formed by introduction of Pro at different sites in CcdB and the nonbonded energy due to these short contacts were calculated using XTOPROMAKE (as described in Materials and Methods) and examined for their correlation with Pro mutant activity data. Only at six positions (residues 10, 11, 43, 44, 53, and 55) was it possible to introduce Pro with small or negligible steric hindrance. Of these six positions, Pro mutants were experimentally available at four positions (residues 10, 11, 43, and 55). At all four positions, mutants were soluble and showed a WT-like phenotype. All other residues showed unfavorable nonbonded energy upon Pro substitution, and at 23 sites the Pro coordinates could not be geometrically fixed. These results were not consistent with experimental data as Pro was tolerated at 45% and 74% of residues in CcdB at the lowest and highest expression levels, respectively. We purified two of the mutants 10P and 43P, which were predicted to have a small number of short contacts, for further thermodynamic characterization. We also purified 101P. Residue 101 is adjacent to a Gly residue at position 100. The presence of a flexible Gly residue preceding Pro should permit the necessary main chain rearrangements required to accommodate Pro. Both 10P and 43P showed an active phenotype at 0% arabinose. 101P showed an inactive phenotype at both 0% and 0.1% arabinose, because it is a known active site residue [27]. The corresponding Ala mutant is also inactive [13]. Equilibrium unfolding studies using GdnCl were carried out for WT and these three mutants, and data was analyzed using a global fit with a common m value (Figure S1). Unfolding parameters ΔG_u_° (free energy change upon protein unfolding at zero denaturant concentration) and C_m_ (denaturant concentration at which fraction of unfolded protein is 0.5) obtained from these denaturation studies are listed in the Figure S1 caption. 10P and 43P showed a 9% decrease in ΔG_u_° while 101P had identical stability to WT. The above results demonstrate that while the XTOPROMAKE program correctly identifies a few nonperturbing sites, it fails to identify the majority of such sites. Hence, mutant models were generated by a procedure that minimizes the overall energy of the protein by rearranging a backbone and side chain using the program MODELLER.

### Correlation of Activity with Structural Parameters

Attempts were made to correlate the activity data with various structural parameters related to the WT protein and/or the Pro mutant models. Figures 2 and S2 show some correlations between the activity of the Pro mutant at each residue position and various structural parameters calculated from either WT native (crystal structure 3vub) or mutant model structures. Five models of each mutant were constructed and the average value of each of the structural parameters was calculated. Pro mutants of the seven active site residues (see earlier secondary structure section) were not considered in this study. Correlation of activity of Pro mutants with the following structural parameters were examined (Figure 2): a) WT residue ACC, b) depth, c) |φ(WT) − (−65°)|, d) solubility, and e) whether WT main chain amide is H-bonded to another protein atom and if WT amide is H-bonded, whether the corresponding acceptor is H-bonded in a mutant model. The statistical significance of correlation for parameters a)–c) was assessed by a nonparametric two-tailed Mann-Whitney test and for parameters d) and e) by Fisher's test using the software GraphPad Prism. *p*-Values in all cases were <0.05, showing that the activity data and the structural parameters are significantly correlated. While most of the nonperturbing mutants were at residues with higher ACC and lower depth than perturbing mutants (Figure 2A and 2B), it was not possible to apply an ACC cutoff to distinguish between perturbing and nonperturbing mutants. However, for most of the nonperturbing mutants, the φ value of the WT residue was close to the PDB average Pro φ value of (−65° ± 15°), and in several of the perturbing mutants |φ(WT) − (−65°)| was >15°. Most perturbing mutants were insoluble (Figure 2D). There was also a significant correlation observed between activity and H-bonding of the amide proton of the WT residue. Twenty-six out of 35 nonperturbing mutants did not have the main chain amide involved in H-bonding, and 26 of 30 residues where the WT main chain amide is not H-bonded (class 1, Figure 2E) were active. For 28 out of 35 perturbing mutants, the main chain amide of the WT residue was H-bonded to another protein atom, and 31 of 40 residues where the WT main chain amide is H-bonded were inactive (Figure 2E). Additional parameters examined are shown in Figure S2 as follows: a–c) mutant Pro contact area ACC (total, main chain only, side chain only, respectively), d) MODELLER objective function value, e) average φ of mutant Pro, f) Average ψ of mutant Pro, g) |φ(WT) − φ(mut)|, h) |ψ (WT) − ψ (mut)|, i) RMSD (φ(WT) − φ (mut)), j) RMSD (ψ (WT) − ψ (mut)) for an 11-residue window centered at the position of mutation, and k) number of neighboring residues. The two-tailed Mann-Whitney test yielded *p*-values less than 0.0001 only for the accessibility data (a–c) and *p*-values less than 0.05 for the φ(WT) − φ(mut), ψ(WT) − ψ(mut), and Ngh(WT) − Ngh(mut) data (g,h,k). The remaining structural parameters did not show a clear correlation with activity data. In the present studies, we did not observe any preference for particular amino acid residues to precede nonperturbing Pro mutants.

### Decision Tree to Predict Effect of Pro on Protein Structure and Activity

**Figure 3:**
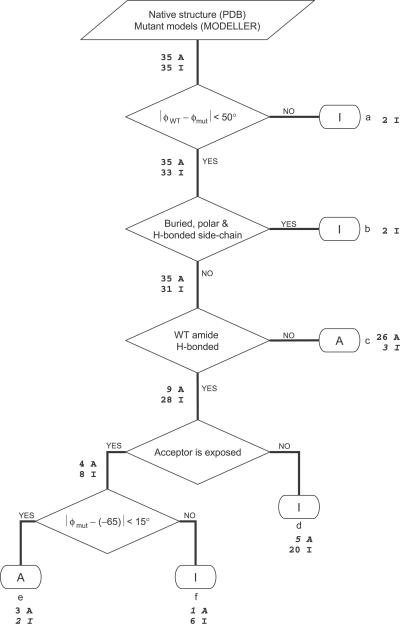
Model Decision Tree The flowchart of scheme for prediction of effects of Pro mutations on protein stability derived from structural analysis of WT crystal structure and mutant models. A and I refer to active and inactive predictions, respectively. Letters in lowercase (a–f) refer to each node of the scheme as described in the text. Numbers at each branch indicate the number of active and inactive mutants satisfying the “Yes” or “No” criteria of the respective branch. The numbers at each A and I prediction bubble correspond to the number of CcdB Pro mutants ending at that bubble. The numbers of misclassified mutants are shown in italics. Active mutants correspond to nonperturbing Pro substitutions and inactive ones to perturbing Pro substitutions in case of CcdB.

**Figure 4:**
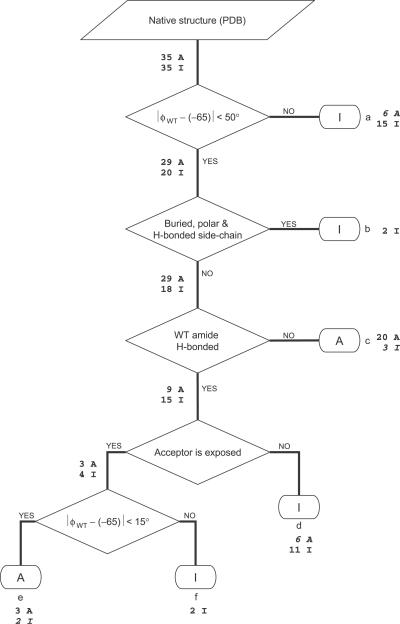
WT Decision Tree The flowchart of scheme for prediction of effects of Pro mutations on protein stability derived from structural analysis of WT-CcdB crystal structure alone. A and I refer to active and inactive predictions, respectively. The numbers at each branch indicate the number of active and inactive mutants satisfying the “Yes” or “No” criteria of the respective branch. The numbers at each A and I prediction bubble correspond to the number of CcdB Pro mutants ending at that bubble. The numbers of misclassified mutants are shown in italics.

A significant correlation of the perturbing/nonperturbing nature of the CcdB Pro mutants was observed primarily with the φ value and H-bonding of the WT amide NH group. A decision tree (Figure 3) was generated taking into account these two correlations to discriminate between active and inactive mutants. Five nodes were defined in this model decision tree based on the following criteria: a) inactive, if |φ(wt) − φ(mut)| > 50° as large main chain rearrangements are likely to be associated with a significant energetic penalty; b) inactive, if WT residue has H-bonded, buried polar side chain as the replacement of a buried polar side chain with Pro will result in unsatisfied H-bond acceptors/donors; c) active, if WT amide NH group is not H-bonded; d) inactive, if acceptor of WT amide H-bond is buried in mutant models. The acceptor could be either main chain or side chain depending on the location of the acceptor atom and is considered as buried if the corresponding average accessibility from five mutant models is <5%; e) active, if acceptor of WT amide H-bond is exposed in mutant models (solvent-exposed acceptor can form H-bond with a water molecule) and |φ(mut) − (−65)|< 15° (since the difference between φ(mut) and average Pro φ is within 15° little energetically unfavorable main chain rearrangements are expected); f) inactive, if acceptor of WT amide H-bond is exposed and |φ(mut) – (−65°)|> 15°. The number of active and inactive CcdB mutants satisfying each of the criteria is also indicated in Figure 3. Out of 35 nonperturbing mutants, 29 were predicted correctly as active/nonperturbing (true positives, TP), and six were incorrectly predicted as perturbing (false negatives, FN), whereas out of 35 perturbing mutants, 30 were correctly predicted as inactive/perturbing (true negatives, TN) and five were predicted as nonperturbing (false positives, FP). The accuracy is defined as a fraction of total correct predictions, (TP + TN) / (TP + TN + FP + FN). The accuracy of the model decision tree is therefore 84% for CcdB activity data (with active site and WT Pro residues excluded). The accuracy drops slightly to 81% if active site residues are also considered. Of the seven Pro mutants at active site residues, three are correctly predicted as inactive. To examine if it was possible to obtain accurate phenotypic predictions in the absence of mutant models, a second (WT) decision tree was considered (Figure 4). This was closely based on the model decision tree (Figure 3) with differences primarily localized to nodes a, e, and f. At node a, since φ(mut) is not available, instead of |φ(wt) − φ(mut)| the value of |φ(wt) − (−65)| is calculated, assuming that the actual value of φ(mut) will be close to −65°. Similarly, at nodes e and f, since φ(mut) is not available, the value of φ(wt) is used instead. This WT decision tree has an accuracy of about 76% (TP = 23, TN = 30, FP = 5, FN = 12), and here the accuracy remains approximately the same (75%) if active site mutants are included. Both the decision trees accurately predicted the nonperturbing nature of Pro at all positions where the WT residue was Pro. Thus, in the case of CcdB, using structural parameters from mutant modeled proteins is somewhat more accurate than using just the native structure in predicting the effect of Pro substitution, although the WT decision tree also gives satisfactory predictions.

Since Pro can potentially occur in either a cis or a trans conformation, cis Pro mutant models were built in addition to the trans Pro mutant models at all residue positions. The only potential benefit of models with cis Pro residues would be in cases where the trans Pro residues were predicted as inactive, while the prediction conferred activity to models with cis Pro mutants. No such cases exist for the present CcdB dataset. The large conformational changes associated with introduction of cis Pro make reliable modeling of this residue difficult. Coupled with the lack of significant improvement in prediction accuracy upon incorporation of cis Pro, this suggests that it is not appropriate to include cis Pro models into the current prediction scheme at the present time.

### Lysozyme Data Analysis

To validate the decision trees described above, they were applied to predict effects of Pro mutations on the activity of T4 lysozyme. In a previous study [28], each of the 163 codons of T4 lysozyme was individually replaced by an amber stop codon. The resulting mutant plasmids were transformed into 13 different suppressor strains, one of which incorporated Pro in place of the stop codon. Plaque-forming phenotypes of these mutants were reported at both 25 and 37 °C. Phenotypic data acquired from suppressor strains have some limitations because suppression efficiency is variable and context-dependent. Nevertheless, this is a large independent dataset acquired with different experimental methodology on a different protein and therefore useful for evaluating the decision trees. This dataset contains 110 active and 53 inactive mutants at 37 °C and 121 active and 42 inactive mutants at 25 °C (Table S2). The model decision tree works reasonably well with an accuracy of 77% with 37 °C data (TP = 84, TN = 41, FP = 12, FN = 26), whereas the WT decision tree yields an accuracy of 73% (TP = 76, TN = 43, FP = 10, FN = 34). Similar results were also obtained when the model and WT decision trees were applied to the phenotypic data acquired at 25 °C. The model decision tree has an accuracy of 74% (TP = 87, TN = 33, FP = 9, FN = 34), whereas the WT decision tree has an accuracy of 70% (TP = 79, TN = 35, FP = 7, FN = 42).

### SNP Data Analysis

There are about 400,000 known nonsynonymous single nucleotide polymorphisms (nsSNPs) in the protein coding sequence of the human genome [29]. Prediction of their functional effects is a crucial aspect of current genomic science. An nsSNP can alter protein function by changing the stability of its native structure and/or its binding properties. Several studies have attempted to predict the functional effects of uncharacterized nsSNPs using empirically derived rules that distinguish disease-associated SNPs and neutral SNPs. These rules were based on 3D structural parameters, sequence-based properties, and multiple alignment of homologous sequences [30–37]. The strongest correlations of perturbing nsSNPs are observed with structural parameters such as packing, H-bonds, and residue solvent accessibility. Approximately, 70%–80% of disease-associated nsSNPs could be explained using features of protein structure. One problem with previous studies is the paucity of validated negative controls, i.e., nsSNPs that definitely do not perturb protein stability/function. Therefore, these programs predict a large number of false positives (10%–30%) [33,36]. Most prior studies of nsSNPs have considered all types of substitutions and were based on structural parameters derived from analyzing the WT native structure. Such an approach does not take into account changes in protein structure that may occur to accommodate the mutation. Pro has unique conformational properties and a rigid structure. Hence, modeling and prediction of functional consequences of Pro containing nsSNPs is qualitatively different from those of other nsSNPs. In the present work, we have generated a decision tree to predict effects of Pro substitution based on our experimental studies on CcdB. About 8% of 14,250 disease-associated nsSNPs (listed at http://ca.expasy.org/cgi-bin/lists?humsavar.txt) involve Pro substitutions. However, in many of these, the structure of the region of the protein containing the Pro mutation had not been determined. Single nucleotide substitutions of the following seven amino acid codons can potentially result in introduction of Pro: Leu, Ser, Thr, Ala, His, Gln, and Arg. We extracted 74 Pro disease-associated nsSNPs in 17 proteins (with known 3D structure) from the above SNP database to evaluate our algorithm. Five mutant models were generated for each of these 17 proteins having a Pro substitution at positions mentioned in Table S3. Mutants were assessed as perturbing or nonperturbing using the decision tree (Figure 3). The perturbing nature of the Pro nsSNPs could be correctly predicted in 56 out of 74 cases, i.e., 76% accuracy (TP = 0, TN = 56, FP = 18, FN = 0). In comparison, accuracy of WT decision tree was 77% (TP = 0, TN = 57, FP = 17, FN = 0). In seven of the cases in Table S3 (examples 5, 24, 45, 46, 47, 61, 63), we misclassified disease-associated nsSNPs as nonperturbing. This was because the acceptor of the amide NH of WT residue was observed to be exposed and the mutant models did not show significant main chain rearrangements from the average Pro φ value (verbar;φ(mut) − (−65)| < 15°). In 11 of the remaining cases in Table S3 (examples 9, 10, 16, 20, 32, 39, 41, 50, 55, 60, and 72), |φ(mut) − φ(WT)| < 50° (average value was ∼12° for these residues) and the WT amide NH group was also not involved in H-bonding. Hence these mutants were predicted to be nonperturbing even though the nsSNPs were associated with diseases. It should be noted that for the disease-associated nsSNPs we have not incorporated any active site information. For example, four of the CcdB Pro mutants at active site positions (residues 24, 25, 95, and 101) were predicted incorrectly as nonperturbing using the decision tree. If any of the Pro containing nsSNPs are at active/functional sites, the activity will be altered even if Pro has been accommodated without perturbing the overall structure/stability of the protein. Moreover, for many of the nsSNPs, the correlation with disease is based on small-size population-based studies and no functional characterization has been done. Hence in at least some of the cases the nsSNPs may actually be nonperturbing, even though they have been classified as disease-associated.

### ProTherm Data Analysis

The algorithm was also assessed using Pro substitutions from the ProTherm database (http://gibk26.bse.kyutech.ac.jp/jouhou/protherm/protherm_search.html) and literature [38–40]. We analyzed 52 Pro mutants corresponding to 19 different proteins for which thermodynamic parameters for stability changes are either reported in the ProTherm database or are taken from the literature (Table S4). A Pro substitution was defined as perturbing if T_m_ (mutant) − T_m_ (WT) was < −10 °C or ΔG(mutant) − ΔG(WT) < −0.5 kcal/mol where T_m_ and ΔG are the temperature at midpoint of thermal unfolding and free energy of unfolding, respectively. Our predictions were correct in 37 out of 52 cases (accuracy is 71%, TP = 32, TN = 5, FP = 4, FN = 11). In comparison, the accuracy of WT decision tree was 69% (TP = 30, TN = 6, FP = 3, FN = 13).

The overall prediction results for all datasets in terms of accuracy, precision, and recall are summarized in Table 3. Precision is the ratio of the correctly identified positives to all positives identified (TP) / (TP + FP), and recall is the ratio of the correctly identified positives to all positives (TP) / (TP + FN). The accuracy and recall values are reasonably high for all the datasets tested except for nsSNPs. In this case, since only perturbing mutations are available (TP = 0), it is not meaningful to calculate precision and recall values.

**Table 3:**
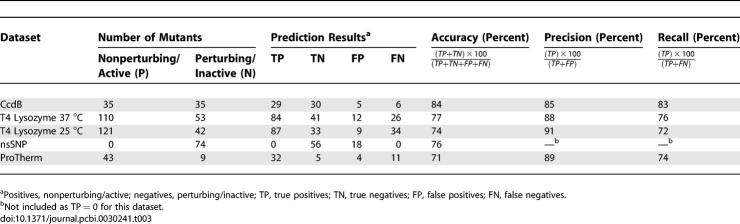
Overall Prediction Results in Terms of Accuracy, Precision, and Recall

### Conclusions

We have constructed a decision tree to predict whether mutating any residue in a protein to Pro will perturb its activity or not. The decision tree uses stereochemical criteria that were derived from protein activity data obtained from a Pro scanning mutagenesis study on CcdB. Predictions were made on 77 Pro mutations in CcdB, 163 Pro mutations in T4 lysozyme, 74 Pro nsSNPs in 17 human proteins, and 52 Pro mutations extracted from the ProTherm database and literature. On average, excluding the CcdB data, the prediction accuracy was 75%. The study also shows that the introduction of Pro within regions of regular secondary structure is not necessarily destabilizing and that introduction of Pro into regions lacking secondary structure can be destabilizing. Hence use of Pro scanning mutagenesis to assign secondary structure has limitations.

Previous studies that predict the effects of nsSNPs on protein function have often employed multiple complex correlations and cannot easily ascribe a physical reason for a prediction. The decision tree described in this study is able to attribute physical cause for the perturbing or nonperturbing nature of a Pro mutation. The essential input required is the crystal structure or an accurate homology model of the WT protein. In most previous studies of predicting the effects of mutations, the lack of nonperturbing mutants has led to a significant degree of overprediction of the negative impact. Our CcdB dataset has an almost equal number of perturbing and nonperturbing mutants, making it ideally suited for benchmarking methods that predict the structural effects of mutations. All of these features make the decision tree described in this study an attractive method for protein engineering and design and to validate and predict the effect of Pro mutations, especially in unannotated Pro nsSNPs of proteins associated with disease. The decision tree when combined with experimental data could also contribute to the evaluation of models of protein structure.

## Materials and Methods

### Plasmids and host strains.

The CcdB gene was cloned under the control of the arabinose inducible P_BAD_ promoter in the vector pBAD24 to yield the construct pBAD24CcdB. In this plasmid, the level of CcdB expression can be regulated by varying the inducer concentration [41]. Three *E.coli* host strains were used: *TOP10*, *XL1 Blue*, and *CSH501*, as described previously [13]. *TOP10* is sensitive to the action of CcdB and used for screening the phenotype. *XL1Blue* is able to tolerate low levels of CcdB protein expression because of the presence of the antidote CcdA, which is encoded by the resident F plasmid, and was used for plasmid propagation. *CSH501* is completely resistant to the action of CcdB because the strain harbors the *GyrA462* mutation in its chromosomal DNA and prevents gyrase from binding to CcdB. *CSH501* was kindly provided by Dr. M. Couturier (Universite Libre de Bruxelles, Belgium) and was used for monitoring expression of mutant proteins.

### Mutagenesis and sequencing.

Thirty-nucleotide-long primers to generate CcdB mutants were designed using OLIGO version 6.0 and were obtained in 96-well format from the PAN Oligo facility at Stanford University. Each residue in CcdB was replaced with Pro using a mega-primer–based method of site-directed mutagenesis as described previously [13,42]. Templates for sequencing to confirm mutations in CcdB were isolated directly from a colony of mutant plasmid transformed in *XL1Blue* and were amplified by rolling circle amplification using phi 29 DNA polymerase as described in [43]. 3′-protected thiophosphate random hexamer primers and yeast pyrophosphates were obtained from Sigma and phi 29 DNA polymerase from New England Biolabs. The entire coding region of CcdB was subjected to automated DNA sequencing. After sequence confirmation, plasmids were isolated from *XL1Blue* grown in 96-deep-well plates.

### Screening of phenotype of CcdB mutants.

Mutant CcdB plasmids were transformed in *TOP10 E. coli* in 96-well format using PCR strips, and activity was assayed by plating 5 μl of transformation mix on square LB-amp plates (120 × 120 mm) placed on 96-well grids in the absence of arabinose at 37 °C [13]. Since active CcdB is toxic to E. coli, only cells transformed with inactive mutants will survive. The phenotype of all mutants that were inactive at 0% arabinose was also examined at 0.001%, 0.01%, and 0.1% of arabinose. Expression level was monitored for all inactive mutants in *CSH501* in the presence of 0.1% arabinose. Cultures were grown in 96-deep-well plates. Following cell lysis by a freeze-thaw method [44], expression and solubility of all Pro mutants of CcdB in *CSH501* was monitored using SDS-PAGE as described previously [13].

### Short contacts and nonbonded energy calculations.

An in-house software, XTOPROMAKE, was used to fix prolyl residues to the backbone at all residue positions of CcdB where the backbone conformation was compatible with closure of the Pro ring. The atoms of the Pro ring, (viz.*,* C^β^, C^γ^, and C^δ^, and their associated hydrogen atoms H^β1^, H^β2^, H^γ1^, H^γ2^, H^δ1^, and H^δ2^) were examined for short contacts with spatial neighbors in the protein structure using the Ramachandran contact criteria [45–47]. In addition, nonbonded van der Waals energy of interaction between these atoms and those which occur within a sphere of 4.0 Å, was computed using standard constants [45]. The choice between *endo* and *exo* configurations of C^γ^ was decided using the energetic criteria. The software ordered the Pro-mutations at all sites, in the order of increasing nonbonded energy arising due to the mutated-prolyl residue. Hence the best sites for Pro introduction could be chosen in conjunction with other criteria (such as H-bonding of the WT residue, accessibility, polarity, etc.). Three Pro mutants which were predicted to have favorable nonbonded energy from XTOPROMAKE were selected for further studies mentioned below.

### Protein purification and thermodynamic characterization.

WT CcdB and three of its Pro mutants (R10P, S43P, and I101P) were purified to homogeneity as described previously [16]. Equilibrium unfolding as a function of GdnCl concentration at 25 °C was monitored by fluorescence spectroscopy at a concentration of 2 μM (dimeric) protein concentration. Fluorescence measurements were done using a SPEX Fluoromax-3 spectrofluorimeter with a 1 cm water-jacketed cell. The excitation and emission wavelengths were fixed at 280 nm and 385 nm, respectively, with slit widths of 2 nm for both excitation and emission monochromators. Each measurement was an average of four readings. The unfolding data was fitted to a two-state unfolding coupled to subunit dissociation model as described earlier [16]. The unfolding data for all three proteins was globally fitted using a single m value.

### Modeling Pro mutants of CcdB.

Five models of each of the CcdB Pro mutants (targets), in trans and cis conformations, were generated by comparative structure modeling using MODELLER 9v1 [48]. MODELLER implements comparative protein structure modeling by satisfaction of spatial restraints that include (i) homology-derived restraints on the distances and dihedral angles in the target sequence, extracted from its alignment with the template structures; (ii) stereochemical restraints such as bond length and bond angle preferences, obtained from the CHARMM-22 molecular mechanics force-field [49]; (iii) statistical preferences for dihedral angles and nonbonded interatomic distances, obtained from a representative set of known protein structures; and (iv) optional manually curated restraints. The spatial restraints, expressed as probability density functions, are combined into an objective function that is optimized by a combination of conjugate gradients and molecular dynamics with simulated annealing. This model-building procedure is similar to structure determination by NMR spectroscopy. The WT–CcdB dimeric structure (PDB code 3vub) was used as template. Target–template alignments are trivially generated by replacing the WT residues by Pro at the position of mutation in a self-alignment of the sequence of 3vub. For each of the mutants, five different models were built from different random initial starting conformations by satisfying the same set of restraints. Models were built using the “automodel” class of MODELLER, with default parameters. For cis Pro mutants, the torsion angle ω was explicitly restrained to a value of 0°. A comprehensive description of comparative protein structure modeling using MODELLER is described in the manual (http://salilab.org/modeller/manual/) and several review articles [48,50,51]. Typically, the five models of the same mutant are all within a 0.5 Å C^α^ RMSD of each other. MODELLER was also used to compute structural properties of the models, including dihedral angles, solvent-accessible surface areas, H-bonds, and residue neighbors. Residue contact accessible surface areas in WT-CcdB and in Pro mutant models were calculated using a probe radius of 1.4 Å. Residue accessibilities for each Pro mutant were averaged over the five models. Main chain dihedral angles of the mutant models (φ and ψ) were similarly averaged. In the five models, the RMSD of the spread of the dihedral angle φ is within 1°. The RMSD of the φ and ψ angles for each residue for an 11-residue window centered around the mutant Pro was computed. The number of neighbors of a residue is the number of residues that have at least one of its atoms within 6 Å of any atom of the residue. H-bonds are detected if the donor–acceptor distance is less than 3.5 Å and the angle donor–acceptor–acceptor antecedent is 120° or greater [52]. The average (in five models) number of H-bonds satisfied by the acceptor (of the amide N in the WT) was calculated. Based on these data, a decision tree was devised to predict the effect (perturbing/nonperturbing) of a Pro substitution at a specified location for any globular protein. Using this algorithm, the activity of CcdB Pro mutants was predicted at 70 nonactive site residue positions mutated. Seven mutants were part of the active site as obtained from Ala and Asp scanning mutagenesis [13] and were therefore excluded from the actual analysis. The accuracy of prediction was calculated by comparison to observed activity data from experiments. Activity was also predicted using another decision tree that was built considering only the WT crystal structure, (i.e., without using mutant models).

### Tests of significance.

A nonparametric two-tailed Mann-Whitney test was performed to assess the significance of correlation between the activity data and various structural parameters using GraphPad Prism (version 5.01 for Windows, GraphPad Software, http://www.graphpad.com). In case of solubility and H-bonding, there are a large number of identical values in the distribution, and hence the Mann-Whitney test could not be used. Instead, Fisher's test was performed to test the association of the parameter and the activity. In all cases, the correlation is considered to be significant if the *p*-value is <0.05.

### Prediction accuracy definitions.

Accuracy is calculated as the ratio of all correct predictions to total predictions, (TP + TN) / (TP + TN + FP + FN) where TP, TN, FP, and FN denote true positives, true negatives, false positives, and false negatives, respectively. Precision is the ratio of the correctly identified positives to all positives identified, i.e., (TP) / (TP + FP), and recall is the ratio of the correctly identified positives to all positives, i.e., (TP) / (TP + FN).

### Lysozyme database analysis.

Five models for each of 163 Pro substitution mutants were generated from the alignment between WT and mutant sequence using MODELLER 9v1 [48]. The WT protein structure (pdb id 2lzm) was used as the template in each case to generate the models. The models were analyzed using the decision tree derived from the CcdB scanning mutagenesis data, and the mutation was predicted to be either active/nonperturbing (P) or inactive/perturbing (N). The correctness of the prediction is judged by comparison with experimental phenotypic activity data.

### SNP analysis.

Seventy-four SNPs with Pro substitutions in 17 different proteins of known 3D structure were selected from the SNP database for validating the algorithm generated from CcdB Pro scanning mutagenesis. Five models for each SNP mutant protein were generated using the WT structure as a template as described above. The models were analyzed using the decision trees as described above and the mutation was predicted to be either perturbing or nonperturbing. If a disease-associated SNP was found to be perturbing, the prediction was assumed to be correct.

### ProTherm database analysis.

52 neutral/stabilizing and destabilizing Pro mutants from 19 different proteins were selected from the ProTherm database and literature, and five models of each mutant were generated using the WT structure as a template as described above. Models were analyzed using the decision trees as described above. Predictions were assumed to be correct if predicted perturbing mutations were experimentally found to be destabilized or if predicted nonperturbing mutations were experimentally found to be neutral or stabilizing.

## Supporting Information

Figure S1 Equilibrium GdnCl Denaturation of CcdB at pH 7.0 Equilibrium GdnCl denaturation profiles of CcdB at pH 7.0 at 25 °C for WT (•), 10P (), 43P (Δ), and 101P (ο). The isothermal melts were carried out in 10 mM HEPES pH 7.0 using 2 μM dimeric protein concentration. The theoretical curves were obtained by fitting all the melts together to a global fit function with a single m (−5.5 kcal mol^−1^ M^−1^) value for two-state unfolding in conjunction with subunit dissociation for dimeric proteins. ΔG^0^ and C_m_ values for WT, 10P, 43P, and 101P were 20.2 ± 0.1, 18.1 ± 0.1, 18.8 ± 0.2, 20.3 ± 0.1 kcal mol^−1^ and 2.4 ± 0.03, 2.1 ± 0.01, 2.2 ± 0.04, 2.4 ± 0.03 M, respectively.
(61 KB PDF)

Figure S2 Correlations of Pro Mutant Activity with Various Structural Parameters of WT and Mutant Models (A) Mutant Pro ACC, (B) mutant Pro main chain ACC, (C) mutant Pro side chain ACC, (D) MODELLER objective function value, (E) average φ of mutant Pro residue, (F) average ψ of mutant Pro residue, (G) average φ difference between WT and mutant Pro, (H) average ψ difference between WT and mutant Pro, (I) φ RMSD for an 11-residue window centered at mutation, (J) ψ RMSD, and (K) average difference in the number of neighboring residues in WT and mutant. Correlations with *p*-values less than 0.05 are considered statistically significant.
(70 KB PDF)

Table S1 Phenotype and Solubility of Pro Mutants of CcdB at 0% and 0.1% Arabinose (151 KB DOC)

Table S2 Assessment of Algorithm Using Proline Substitution Phenotypic Data from T4 Lysozyme (246 KB DOC)

Table S3 Assessment of Algorithm Using Proline nsSNPs of Disease-Associated Proteins All mutants in this dataset are assumed to be perturbing.
(76 KB DOC)

Table S4 Assessment of Algorithm Using Pro Substitutions from ProTherm Database and Literature (110 KB DOC)

## Abbreviations

ΔGu°: free energy change upon protein unfolding at zero denaturant concentration
ACC: percentage side chain solvent accessible surface area of a residue
CcdB: controller of cell division or death B protein
Cm: denaturant concentration at which fraction of unfolded protein is 0.5
H-bond: hydrogen bond
m: cooperativity of denaturant induced unfolding
mut: Mutant
nsSNP: nonsynonymous single nucleotide polymorphism
Pro: proline
TP: true positive
WT: wild type
