# Supplementary material for: Stereochemical Criteria for Prediction of the Effects of Proline Mutations on Protein Stability
Source: PLoS Comput Biol. 2007 Dec 7;3(12):e241. doi: 10.1371/journal.pcbi.0030241 (PMC2134964; doi:10.1371/journal.pcbi.0030241)
Supplement: Supplementary file 1 [file pcbi.0030241.sg001.pdf]

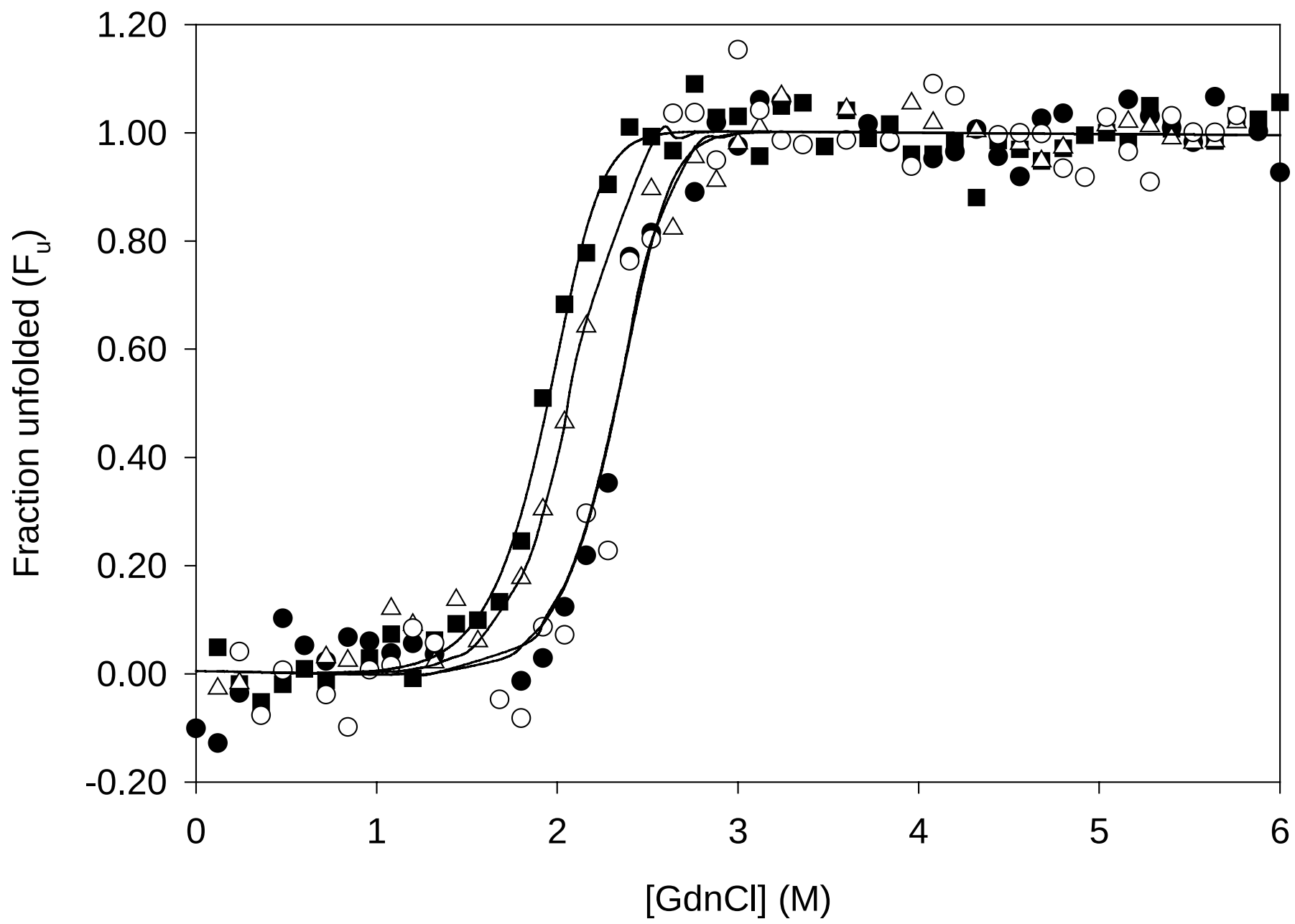

1.20
1.00
0.80
0.60
0.40
0.20
0.00
-0.20
Fraction unfolded (F_u)
0
1
2
3
4
5
6
[GdnCl] (M)
