# Supplementary material for: Stereochemical Criteria for Prediction of the Effects of Proline Mutations on Protein Stability
Source: PLoS Comput Biol. 2007 Dec 7;3(12):e241. doi: 10.1371/journal.pcbi.0030241 (PMC2134964; doi:10.1371/journal.pcbi.0030241)
Supplement: Supplementary file 2 [file pcbi.0030241.sg002.pdf]

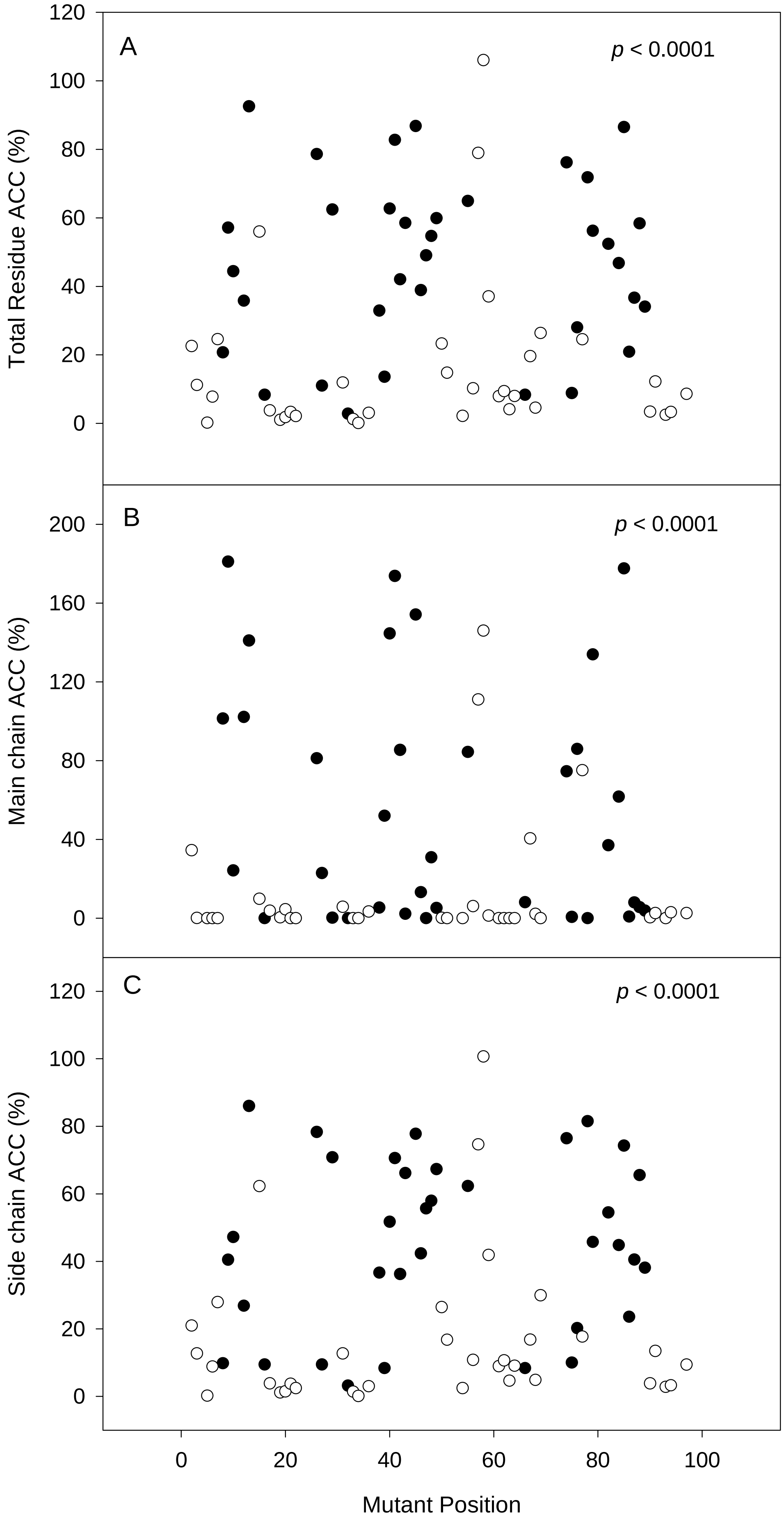

A
p < 0.0001
Total Residue ACC (%)
120
100
80
60
40
20
0
B
p < 0.0001
Main chain ACC (%)
200
160
120
80
40
0
C
p < 0.0001
Side chain ACC (%)
120
100
80
60
40
20
0
0
20
40
60
80
100
Mutant Position

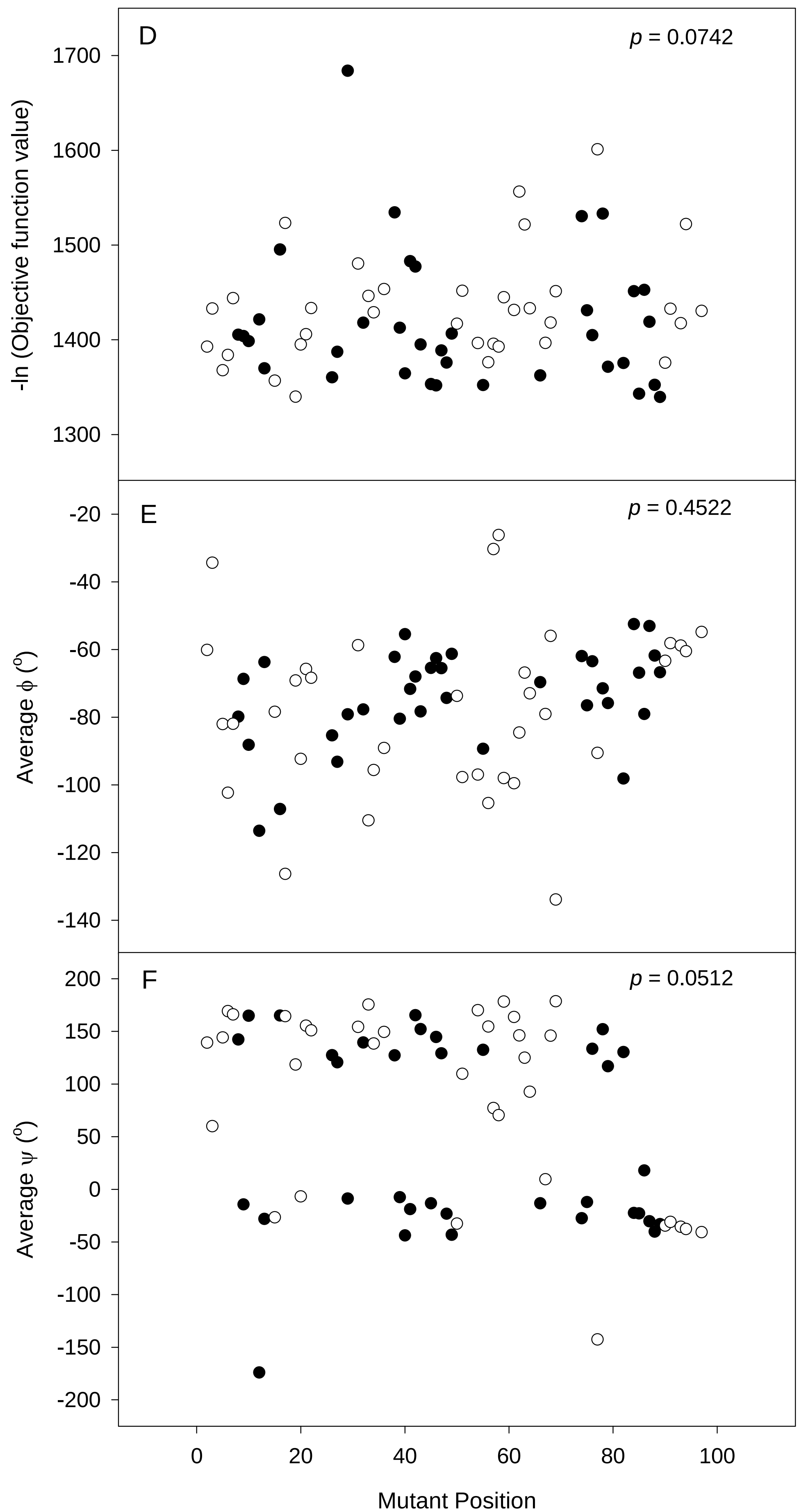
D
p = 0.0742
-ln (Objective function value)
1700
1600
1500
1400
1300
E
p = 0.4522
Average φ (°)
-20
-40
-60
-80
-100
-120
-140
F
p = 0.0512
Average ψ (°)
200
150
100
50
0
-50
-100
-150
-200
0
20
40
60
80
100
Mutant Position

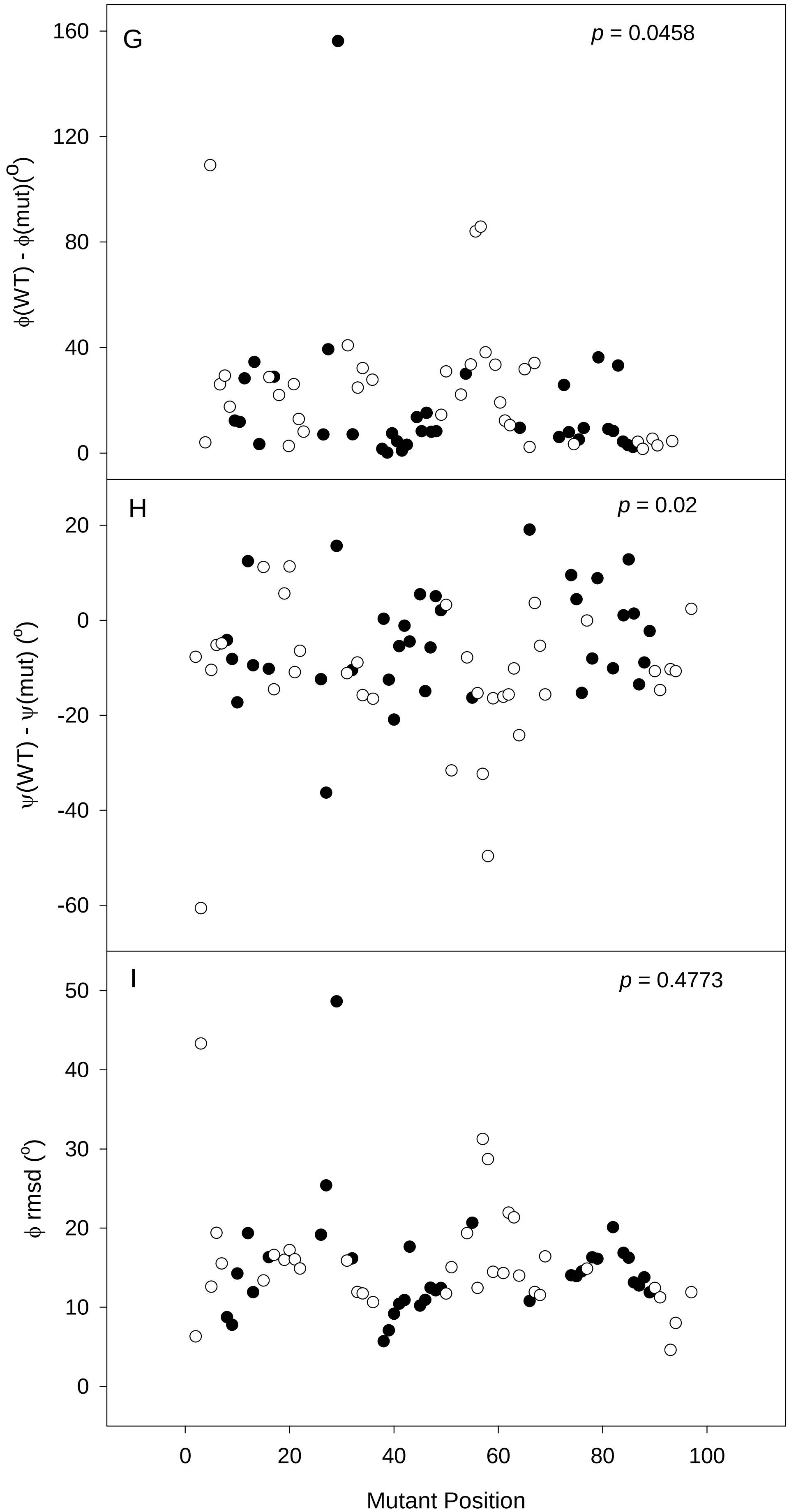

G
p = 0.0458
ϕ(WT) - ϕ(mut)(°)
160
120
80
40
0
H
p = 0.02
ψ(WT) - ψ(mut) (°)
20
0
-20
-40
-60
I
p = 0.4773
ϕ rmsd (°)
50
40
30
20
10
0
0
20
40
60
80
100
Mutant Position

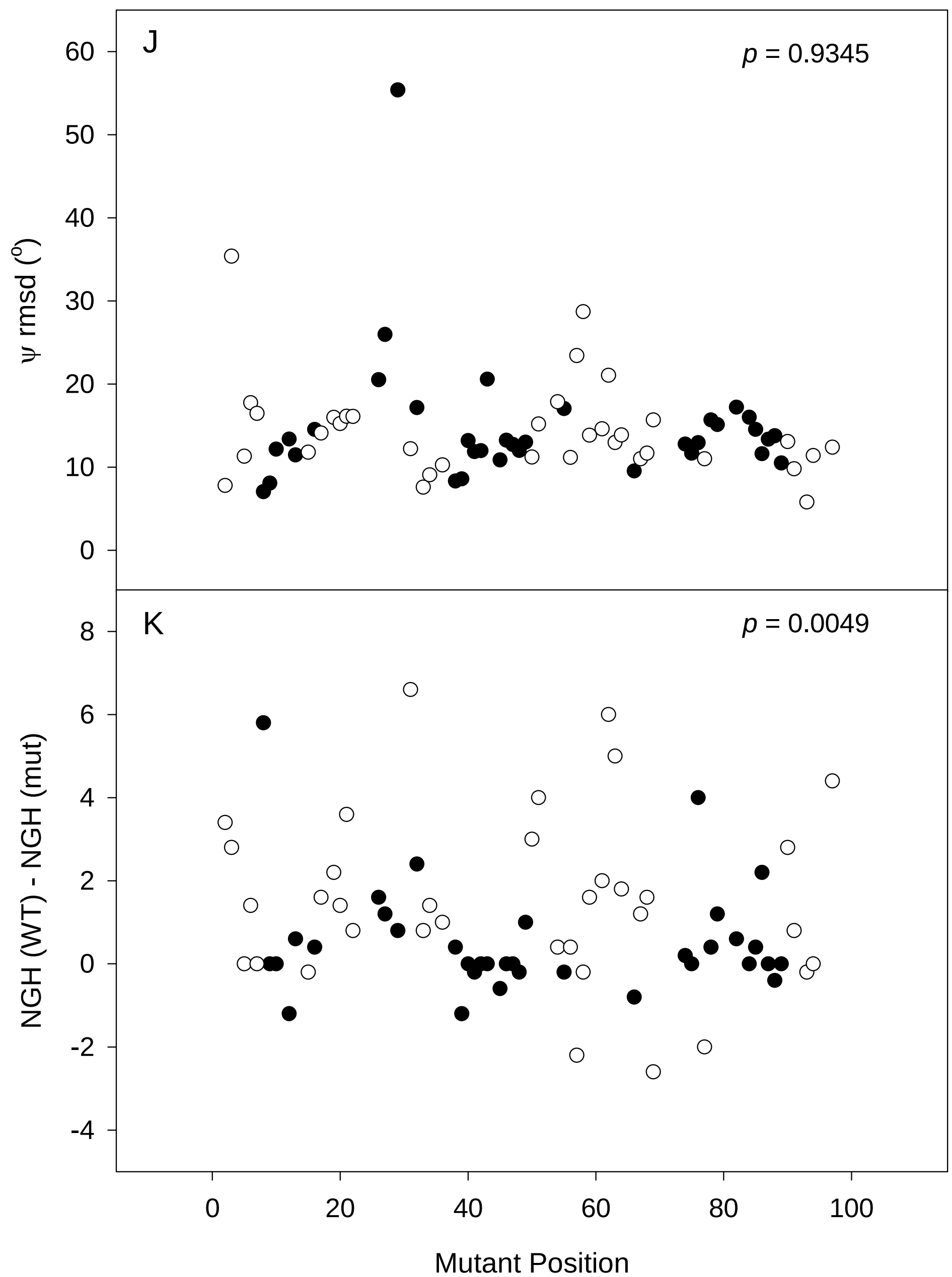
J
p = 0.9345
ψ rmsd (°)
60
50
40
30
20
10
0
K
p = 0.0049
NGH (WT) - NGH (mut)
8
6
4
2
0
-2
-4
0
20
40
60
80
100
Mutant Position
