# Supplementary material for: Stereochemical Criteria for Prediction of the Effects of Proline Mutations on Protein Stability
Source: PLoS Comput Biol. 2007 Dec 7;3(12):e241. doi: 10.1371/journal.pcbi.0030241 (PMC2134964; doi:10.1371/journal.pcbi.0030241)
Supplement: Supplementary file 3 [file pcbi.0030241.st001.doc]

Supplementary Table 1: Phenotype and solubility of Pro mutants of CcdB at 0% and 0.1% arabinose.

N

M

L

| Res No. | WT Res | Activity  (0% ara) | Activity (0.1% ara) | ACC (%) | Sec structure | Predicted Sec Structure ^a^ | Predicted Sec Structure ^b^ | Solubility ^c^ | Activity prediction ^d^ |
| --- | --- | --- | --- | --- | --- | --- | --- | --- | --- |
| 1 | M |  |  | 77 |  |  |  |  |  |
| 2 | Q | I | I | 17 | O | Y | Y | Pe | A |
| 3 | F | I | A | 12 | O | Y | Y | Pe | I |
| 4 | K |  |  | 31 | E |  |  |  | A |
| 5 | A | I | A | 0 | E | Y | Y | Pe | I |
| 6 | Y | I | I | 9 | E | Y | Y | Pe | I |
| 7 | T | I | A | 34 | E | Y | Y | Pe | I |
| 8 | Y | A | A | 11 | E | N | N | Su | A |
| 9 | K | A | A | 64 | O | N | N | Su | I |
| 10 | R | A | A | 77 | O | N | N | Su | A |
| 11 | E |  |  | 111 | O |  |  |  | A |
| 12 | S | A | A | 43 | O | N | N | Su | A |
| 13 | R | A | A | 84 | O | N | N | Su | A |
| 14 | Y |  |  | 17 | O |  |  |  | A |
| 15 | R | I | A | 74 | O | Y | N | Su | A |
| 16 | L | A | A | 11 | E | N | Y | Su | A |
| 17 | F | I | I | 0.2 | E | Y | Y | Pe | I |
| 18 | A |  |  | 0 | E |  |  |  | I |
| 19 | D | I | A | 2 | E | Y | Y | Su | I |
| 20 | A | I | A | 0 | O | Y | Y | Su | I |
| 21 | Q | I | I | 1 | O | Y | Y | Pe | I |
| 22 | S | I | A | 9 | O | Y | Y | Su | I |
| 23 | D |  |  | 60 | O |  |  |  | A |
| 24 | I | I | A | 66 | O | Ac | Ac | Su | A |
| 25 | I | I | A | 15 | O | Ac | Ac | Su | A |
| 26 | D | A | A | 92 | O | N | N | Su | A |
| 27 | T | A | A | 10 | O | N | N | Su | A |
| 28 | P | WT | WT | 80 | O |  |  |  | A |
| 29 | G | A | A | 66 | O | N | N | Su | A |
| 30 | R |  |  | 46 | E |  |  |  | I |
| 31 | R | I | I | 17 | E | Y | N | Su | I |
| 32 | M | A | A | 0.4 | E | N | Y | Su | I |
| 33 | A | I | I | 1 | E | Y | Y | Pe | I |
| 34 | I | I | I | 0 | E | Y | Y | Pe | I |
| 35 | P | WT | WT | 9 | E |  |  |  | A |
| 36 | L | I | I | 0 | E | Y | Y | Pe | I |
| 37 | A |  |  | 12 | E |  |  |  | I |
| 38 | S | A | A | 40 | E | N | N | Su | A |
| 39 | A | A | A | 14 | O | N | N | Su | I |
| 40 | R | A | A | 80 | O | N | N | Su | A |
| 41 | L | A | A | 99 | O | N | N | Su | A |
| 42 | L | A | A | 37 | O | N | N | Su | A |
| 43 | S | A | A | 67 | O | N | N | Su | A |
| 44 | D |  |  | 102 | O |  |  |  | A |
| 45 | K | A | A | 93 | O | N | N | Su | A |
| 46 | A | A | A | 49 | O | N | N | Su | A |
| 47 | S | A | A | 58 | O | N | N | Su | A |
| 48 | R | A | A | 68 | O | N | N | Su | A |
| 49 | E | A | A | 76 | O | N | N | Su | A |
| 50 | L | I | A | 11 | O | Y | Y | Pe | A |
| 51 | Y | I | A | 17 | O | Y | Y | Pe | I |
| 52 | P | WT | WT | 3 | O |  |  |  | A |
| 53 | A |  |  | 25 | E |  |  |  | A |
| 54 | A | I | I | 0.1 | E | Y | Y | Pe | I |
| 55 | H | A | A | 66 | E | N | Y | Su | A |
| 56 | I | I | A | 13 | E | Y | Y | Pe | I |
| 57 | G | I | I | 124 | O | Y | Y | Su | A |
| 58 | D | I | I | 119 | O | Y | Y | Su | I |
| 59 | E | I | A | 36 | E | Y | Y | Pe | I |
| 60 | S |  |  | 38 | E |  |  |  | A |
| 61 | W | I | A | 13 | E | Y | Y | Su | I |
| 62 | R | I | A | 7 | E | Y | Y | Pe | I |
| 63 | M | I | A | 0.1 | E | Y | Y | Pe | I |
| 64 | M | I | I | 22 | O | Y | Y | Pe | I |
| 65 | T |  |  | 0 | O |  |  |  | I |
| 66 | T | A | A | 13 | O | N | N | Su | A |
| 67 | D | I | I | 34 | O | Y | Y | Pe | I |
| 68 | M | I | A | 0 | O | Y | Y | Pe | A |
| 69 | A | I | A | 45 | E | Y | Y | Pe | I |
| 70 | S |  |  | 7 | E |  |  |  | A |
| 71 | A |  |  | 14 | E |  |  |  | I |
| 72 | P | WT | WT | 36 | E |  |  |  | A |
| 73 | A |  |  | 61 | O |  |  |  | A |
| 74 | S | A | A | 86 | O | N | N | Su | A |
| 75 | A | A | A | 7 | O | N | N | Su | I |
| 76 | I | A | A | 17 | E | N | N | Su | A |
| 77 | G | I | I | 7 | E | Y | N | Pe | I |
| 78 | E | A | A | 91 | E | N | N | Su | A |
| 79 | E | A | A | 43 | E | N | N | Su | A |
| 80 | A |  |  | 36 | E |  |  |  | I |
| 81 | A |  |  | 33 | E |  |  |  | A |
| 82 | D | A | A | 48 | E | N | N |  | A |
| 83 | L |  |  | 1 | O |  |  |  | I |
| 84 | S | A | A | 60 | O | N | N | Su | A |
| 85 | H | A | A | 67 | O | N | N | Su | A |
| 86 | R | A | A | 25 | O | N | N | Su | I |
| 87 | E | A | A | 47 | H | N | N | Su | A |
| 88 | N | A | A | 88 | H | N | N | Su | A |
| 89 | D | A | A | 51 | H | N | N | Su | I |
| 90 | I | I | A | 0.1 | H | Y | Y | Pe | I |
| 91 | K | I | A | 42 | H | Y | Y | Pe | I |
| 92 | N |  |  | 64 | H |  |  |  | A |
| 93 | A | I | A | 0 | H | Y | Y | Pe | I |
| 94 | I | I | I | 0.6 | H | Y | Y | Pe | I |
| 95 | N | I | I | 39 | H | Ac | Ac | Pe | A |
| 96 | L |  |  | 35 | H |  |  |  | A |
| 97 | M | I | A | 0 | H | Y | Y | Pe | I |
| 98 | F | I | I | 0.8 | H | Ac | Ac | Pe | I |
| 99 | Y | I | I | 19 | H | Ac | Ac | Su | I |
| 100 | G | I | I | 3 | O | Ac | Ac | Su | I |
| 101 | I | I | I | 125 | O | Ac | Ac | Su | A |

^a^ Secondary structure assignments based on individual residue phenotypes from Proline scanning mutagenesis. Inactive mutants were assigned to be present in regions of secondary structure and active mutants to be in regions of non-secondary structure (Blank spaces indicate that the mutant was not made).

^b^ Secondary structure assignments based on average phenotype in a three-residue window (Blank spaces indicate that the mutant was not made).

^c^ Solubility of CcdB Pro mutants is examined in CSH501 in the presence of 0.1% arabinose.

A and I refer to active and inactive mutants respectively. Su and Pe indicate mutant is in supernatant or pellet respectively after cell lysis. Blank cells indicate mutant was not made. E, H and O refer to residue positions present in extended beta-strand, helix and non-secondary structure of CcdB respectively. Y refers to residue positions which were predicted to be in secondary structure regions (either in H or in E). N refers to residue positions which were predicted to be in non-secondary structure regions (O). Ac refers to residue positions where secondary structural assignments were not done as proline mutations were at active site residues.

^d^ Activity prediction according to model decision tree (Figure 3). Incorrect predictions are shown in red.
