# Supplementary material for: Stereochemical Criteria for Prediction of the Effects of Proline Mutations on Protein Stability
Source: PLoS Comput Biol. 2007 Dec 7;3(12):e241. doi: 10.1371/journal.pcbi.0030241 (PMC2134964; doi:10.1371/journal.pcbi.0030241)
Supplement: Supplementary file 4 [file pcbi.0030241.st002.doc]

Supplementary Table 2. Assessment of algorithm using proline substitution phenotypic data from T4 lysozyme [1]

| Residue No | Residue | 37 °C | | 25 °C | |
| --- | --- | --- | --- | --- | --- |
|  |  | Phenotype^a^ | Correct prediction | Phenotype^a^ | Correct prediction |
| 2 | N | A | Yes | A | Yes |
| 3 | I | A | Yes | A | Yes |
| 4 | F | A | Yes | A | Yes |
| 5 | E | A | No | A | No |
| 6 | M | A | No | A | No |
| 7 | L | I | No | I | No |
| 8 | R | A | Yes | A | Yes |
| 9 | I | A | Yes | A | Yes |
| 10 | D | I | Yes | I | Yes |
| 11 | E | I | Yes | I | Yes |
| 12 | G | I | Yes | I | Yes |
| 13 | L | A | Yes | A | Yes |
| 14 | R | A | No | A | No |
| 15 | L | A | Yes | A | Yes |
| 16 | K | A | Yes | A | Yes |
| 17 | I | A | Yes | A | Yes |
| 18 | Y | I | Yes | I | Yes |
| 19 | K | A | Yes | A | Yes |
| 20 | D | I | Yes | A | No |
| 21 | T | A | Yes | A | Yes |
| 22 | E | A | Yes | A | Yes |
| 23 | G | A | Yes | A | Yes |
| 24 | Y | A | Yes | A | Yes |
| 25 | Y | A | Yes | A | Yes |
| 26 | T | I | Yes | I | Yes |
| 27 | I | I | Yes | I | Yes |
| 28 | G | I | Yes | I | Yes |
| 29 | I | A | Yes | A | Yes |
| 30 | G | I | No | I | No |
| 31 | H | I | Yes | I | Yes |
| 32 | L | A | Yes | A | Yes |
| 33 | L | A | No | A | No |
| 34 | T | A | No | A | No |
| 35 | K | A | Yes | A | Yes |
| 36 | S | A | Yes | A | Yes |
| 37 | P | A | Yes | A | Yes |
| 38 | S | A | Yes | A | Yes |
| 39 | L | A | Yes | A | Yes |
| 40 | N | A | Yes | A | Yes |
| 41 | A | A | Yes | A | Yes |
| 42 | A | A | No | A | No |
| 43 | K | A | Yes | A | Yes |
| 44 | S | A | Yes | A | Yes |
| 45 | E | A | Yes | A | Yes |
| 46 | L | I | Yes | A | No |
| 47 | D | A | Yes | A | Yes |
| 48 | K | A | Yes | A | Yes |
| 49 | A | A | No | A | No |
| 50 | I | I | Yes | A | No |
| 51 | G | A | Yes | A | Yes |
| 52 | R | A | No | A | No |
| 53 | N | A | Yes | A | Yes |
| 54 | C | A | Yes | A | Yes |
| 55 | N | A | No | A | No |
| 56 | G | A | Yes | A | Yes |
| 57 | V | A | Yes | A | Yes |
| 58 | I | A | No | A | No |
| 59 | T | A | No | A | No |
| 60 | K | A | Yes | A | Yes |
| 61 | D | A | Yes | A | Yes |
| 62 | E | I | No | A | Yes |
| 63 | A | I | Yes | A | No |
| 64 | E | A | Yes | A | Yes |
| 65 | K | A | Yes | A | Yes |
| 66 | L | I | Yes | I | Yes |
| 67 | F | I | Yes | I | Yes |
| 68 | N | A | Yes | A | Yes |
| 69 | Q | A | Yes | A | Yes |
| 70 | D | I | Yes | I | Yes |
| 71 | V | A | No | A | No |
| 72 | D | A | Yes | A | Yes |
| 73 | A | A | Yes | A | Yes |
| 74 | A | A | No | A | No |
| 75 | V | I | Yes | I | Yes |
| 76 | R | A | Yes | A | Yes |
| 77 | G | A | Yes | A | Yes |
| 78 | I | A | No | A | No |
| 79 | L | A | No | A | No |
| 80 | R | A | Yes | A | Yes |
| 81 | N | A | No | A | No |
| 82 | A | A | Yes | A | Yes |
| 83 | K | A | Yes | A | Yes |
| 84 | L | I | No | I | No |
| 85 | K | A | No | A | No |
| 86 | P | A | Yes | A | Yes |
| 87 | V | A | No | A | No |
| 88 | Y | I | Yes | I | Yes |
| 89 | D | A | No | A | No |
| 90 | S | A | Yes | A | Yes |
| 91 | L | I | Yes | A | No |
| 92 | D | A | Yes | A | Yes |
| 93 | A | A | Yes | A | Yes |
| 94 | V | A | Yes | A | Yes |
| 95 | R | I | No | A | Yes |
| 96 | R | I | No | I | No |
| 97 | C | A | Yes | A | Yes |
| 98 | A | A | No | A | No |
| 99 | L | I | Yes | I | Yes |
| 100 | I | I | Yes | I | Yes |
| 101 | N | I | Yes | I | Yes |
| 102 | M | I | Yes | A | No |
| 103 | V | I | Yes | I | Yes |
| 104 | F | I | Yes | I | Yes |
| 105 | Q | I | Yes | I | Yes |
| 106 | M | I | Yes | I | Yes |
| 107 | G | I | Yes | I | Yes |
| 108 | E | A | Yes | A | Yes |
| 109 | T | A | Yes | A | Yes |
| 110 | G | A | Yes | A | Yes |
| 111 | V | A | No | A | No |
| 112 | A | A | Yes | A | Yes |
| 113 | G | A | Yes | A | Yes |
| 114 | F | I | Yes | I | Yes |
| 115 | T | A | Yes | A | Yes |
| 116 | N | A | Yes | A | Yes |
| 117 | S | I | Yes | I | Yes |
| 118 | L | I | No | A | Yes |
| 119 | R | A | No | A | No |
| 120 | M | A | Yes | A | Yes |
| 121 | L | I | Yes | I | Yes |
| 122 | Q | A | Yes | A | Yes |
| 123 | Q | A | No | A | No |
| 124 | K | A | No | A | No |
| 125 | R | I | Yes | I | Yes |
| 126 | W | A | Yes | A | Yes |
| 127 | D | A | Yes | A | Yes |
| 128 | E | A | Yes | A | Yes |
| 129 | A | I | Yes | A | No |
| 130 | A | A | Yes | A | Yes |
| 131 | V | A | Yes | A | Yes |
| 132 | N | A | No | A | No |
| 133 | L | I | Yes | I | Yes |
| 134 | A | A | Yes | A | Yes |
| 135 | K | A | Yes | A | Yes |
| 136 | S | I | No | I | No |
| 137 | R | I | No | I | No |
| 138 | W | I | No | I | No |
| 139 | Y | A | Yes | A | Yes |
| 140 | N | A | Yes | A | Yes |
| 141 | Q | A | Yes | A | Yes |
| 142 | T | I | Yes | I | Yes |
| 143 | P | A | Yes | A | Yes |
| 144 | N | A | Yes | A | Yes |
| 145 | R | A | Yes | A | Yes |
| 146 | A | A | No | A | No |
| 147 | K | A | Yes | A | Yes |
| 148 | R | A | Yes | A | Yes |
| 149 | V | I | No | I | No |
| 150 | I | I | Yes | I | Yes |
| 151 | T | A | Yes | A | Yes |
| 152 | T | I | Yes | I | Yes |
| 153 | F | I | Yes | I | Yes |
| 154 | R | I | Yes | I | Yes |
| 155 | T | I | Yes | I | Yes |
| 156 | G | I | Yes | A | No |
| 157 | T | I | No | I | No |
| 158 | W | A | Yes | A | Yes |
| 159 | D | A | Yes | A | Yes |
| 160 | A | A | Yes | A | Yes |
| 161 | Y | I | Yes | I | Yes |
| 162 | K | A | Yes | A | Yes |
| 163 | N | A | Yes | A | Yes |
| 164 | L | A | Yes | A | Yes |

^a^ A and I correspond to active (non-perturbing) and inactive (perturbing) phenotypes respectively.

##### References

1. Rennell D, Bouvier SE, Hardy LW, Poteete AR (1991) Systematic mutation of bacteriophage T4 lysozyme. J Mol Biol 222: 67-88.
