# Supplementary material for: Stereochemical Criteria for Prediction of the Effects of Proline Mutations on Protein Stability
Source: PLoS Comput Biol. 2007 Dec 7;3(12):e241. doi: 10.1371/journal.pcbi.0030241 (PMC2134964; doi:10.1371/journal.pcbi.0030241)
Supplement: Supplementary file 5 [file pcbi.0030241.st003.doc]

Supplementary Table 3. Assessment of algorithm using proline nsSNPs of disease associated proteins. All mutants in this dataset are assumed to be perturbing.

| Number | Protein | PDB ID | Mutation | Correct prediction |
| --- | --- | --- | --- | --- |
| 1 | ANDR^a^ | 2AXA | S759P | Yes |
| 2 | ANDR^a^ | 2AXA | S791P | Yes |
| 3 | ANDR^a^ | 2AXA | L830P | Yes |
| 4 | BRCA1^b^ | 1T15 | L1780P | Yes |
| 5 | BRCA1^b^ | 1T15 | L1705P | No |
| 6 | BRCA1^b^ | 1T15 | L1786P | Yes |
| 7 | BRCA1^b^ | 1T15 | A1752P | Yes |
| 8 | P53^c^ | 1TSR | Q144P | Yes |
| 9 | P53^c^ | 1TSR | L194P | No |
| 10 | P53^c^ | 1TSR | S149P | No |
| 11 | P53^c^ | 1TSR | L145P | Yes |
| 12 | Rb^d^ | 1GH6 | H673P | Yes |
| 13 | Rb^d^ | 1GH6 | L657P | Yes |
| 14 | Rb^d^ | 1GH6 | L662P | Yes |
| 15 | Rb^d^ | 1GH6 | Q685P | Yes |
| 16 | CDKN2C^e^ | 1MX6 | A72P | No |
| 17 | HGPRT^f^ | 1Z7G | L40P | Yes |
| 18 | HGPRT^f^ | 1Z7G | A49P | Yes |
| 19 | HGPRT^f^ | 1Z7G | R50P | Yes |
| 20 | CDKN2A^g^ | 1BI7 | L16P | No |
| 21 | CDKN2A^g^ | 1BI7 | A20P | Yes |
| 22 | CDKN2A^g^ | 1BI7 | R24P | Yes |
| 23 | CDKN2A^g^ | 1BI7 | L32P | Yes |
| 24 | CDKN2A^g^ | 1BI7 | L62P | No |
| 25 | CDKN2A^g^ | 1BI7 | R87P | Yes |
| Number | Protein | PDB ID | Mutation | Correct prediction |
| 26 | CDKN2A^g^ | 1BI7 | H98P | Yes |
| 27 | CDKN2A^g^ | 1BI7 | R99P | Yes |
| 28 | CDKN2A^g^ | 1BI7 | A132P | Yes |
| 29 | BTK^h^ | 1BWN | L11P | Yes |
| 30 | BTK^h^ | 1BWN | R28P | Yes |
| 31 | BTK^h^ | 1BWN | T33P | Yes |
| 32 | BTK^h^ | 1BWN | T117P | No |
| 33 | CFTR^i^ | 1XMI | H620P | Yes |
| 34 | CFTR^i^ | 1XMI | L633P | Yes |
| 35 | GLA^j^ | 1R46 | R49P | Yes |
| 36 | GLA^j^ | 1R46 | L89P | Yes |
| 37 | GLA^j^ | 1R46 | L131P | Yes |
| 38 | GLA^j^ | 1R46 | A143P | Yes |
| 39 | NF1^k^ | 1NF1 | R1250P | No |
| 40 | NF1^k^ | 1NF1 | R1276P | Yes |
| 41 | OTC^l^ | 1OTH | L45P | No |
| 42 | OTC^l^ | 1OTH | L63P | Yes |
| 43 | OTC^l^ | 1OTH | A140P | Yes |
| 44 | OTC^l^ | 1OTH | R141P | Yes |
| 45 | OTC^l^ | 1OTH | L201P | No |
| 46 | OTC^l^ | 1OTH | H255P | No |
| 47 | OTC^l^ | 1OTH | S340P | No |
| 48 | OTC^l^ | 1OTH | L341P | Yes |
| 49 | PKLR^m^ | 1LIU | S80P | Yes |
| 50 | PKLR^m^ | 1LIU | R86P | No |
| 51 | PKLR^m^ | 1LIU | A115P | Yes |
| 52 | PKLR^m^ | 1LIU | L155P | Yes |
| 53 | PKLR^m^ | 1LIU | R337P | Yes |
| Number | Protein | PDB ID | Mutation | Correct prediction |
| 54 | VHL^n^ | 1LM8 | R79P | Yes |
| 55 | VHL^n^ | 1LM8 | R82P | No |
| 56 | VHL^n^ | 1LM8 | L89P | Yes |
| 57 | VHL^n^ | 1LM8 | T105P | Yes |
| 58 | VHL^n^ | 1LM8 | R107P | Yes |
| 59 | VHL^n^ | 1LM8 | L118P | Yes |
| 60 | VHL^n^ | 1LM8 | L158P | No |
| 61 | VHL^n^ | 1LM8 | R161P | No |
| 62 | VHL^n^ | 1LM8 | L178P | Yes |
| 63 | VHL^n^ | 1LM8 | L184P | No |
| 64 | VHL^n^ | 1LM8 | L188P | Yes |
| 65 | ARSB^o^ | 1FSU | L321P | Yes |
| 66 | ARSB^o^ | 1FSU | H393P | Yes |
| 67 | ARSB^o^ | 1FSU | L498P | Yes |
| 68 | COL10A1^p^ | 1GR3 | S600P | Yes |
| 69 | COL10A1^p^ | 1GR3 | L614P | Yes |
| 70 | COL10A1^p^ | 1GR3 | S671P | Yes |
| 71 | FGFR2^q^ | 1EV2 | S267P | Yes |
| 72 | FGFR2^q^ | 1EV2 | A337P | No |
| 73 | FGFR2^q^ | 1EV2 | T341P | Yes |
| 74 | FGFR2^q^ | 1EV2 | A344P | Yes |

^a^Androgen receptor/Prostate cancer, ^b^Breast cancer gene 1/Breast cancer, ^c^P53/colon cancer, ^d^Retinoblastoma/Eye tumor, ^e^Cyclin dependent kinase 6 inhibitor (p18-INK6) /Breast cancer, ^f^Hypoxanthine-guanine phosphoribosyltransferase, ^g^Cyclin dependent kinase 6 inhibitor (p16-INK6) / Cutaneous malignant melanoma 2, ^h^Tyrosine-protein kinase BTK/ X-linked agammaglobulinemia type 1, ^i^Cystic Fibrosis Transmembrane receptor/Cystic fibrosis, ^j^Alpha-galactosidase A/ Fabry disease, ^k^Neurofibromatosis-related protein NF-1/ Type 1 neurofibromatosis, ^l^Ornithine carbamoyltransferase, mitochondrial/ Ornithine hyperammonemia due to carbamoyltransferase deficiency, ^m^Pyruvate kinase/ Chronic nonspherocytic hemolytic anemia, ^n^Von Hippel-Lindau disease tumor suppressor/ Von Hippel-Lindau disease, ^o^Arylsulfatase B/ MucOolysaccharidosis type VI, ^p^Collagen alpha-1(X) chain/ Schmid type metaphyseal chondrodysplasia, ^q^Fibroblast growth factor receptor 2/ Crouzon syndrome.
