# Supplementary material for: Stereochemical Criteria for Prediction of the Effects of Proline Mutations on Protein Stability
Source: PLoS Comput Biol. 2007 Dec 7;3(12):e241. doi: 10.1371/journal.pcbi.0030241 (PMC2134964; doi:10.1371/journal.pcbi.0030241)
Supplement: Supplementary file 6 [file pcbi.0030241.st004.doc]

Supplementary Table 4. Assessment of algorithm using Pro substitutions from ProTherm database and literature.

| No | Protein | PDB id | Mutation | ΔT_m_/ΔΔG^#^ | Phenotype^*^ | Correct prediction |
| --- | --- | --- | --- | --- | --- | --- |
| 1 | Barnase^a^ | 1BNI | T6P | -3.08 | I | No |
| 2 | Barnase^a^ | 1BNI | A32P | -4.10 | I | No |
| 3 | Plasminogen activator inhibitor 1^a^ | 1C5G | E350P | -3.50 | A | No |
| 4 | Plasminogen activator inhibitor 1^a^ | 1C5G | E373P | 0.50 | A | Yes |
| 5 | Catabolite activator protein^a^ | 1G6N | S128P | 0.14 | A | No |
| 6 | α-lactalbumin^a^ | 1HFZ | Y103P | -1.10 | A | No |
| 7 | Alcohol dehydrogenase^a^ | 1KEV | A22P | 0.70 | A | Yes |
| 8 | Alcohol dehydrogenase^a^ | 1KEV | S24P | 3.90 | A | Yes |
| 9 | Alcohol dehydrogenase^a^ | 1KEV | T149P | -1.00 | A | No |
| 10 | Alcohol dehydrogenase^a^ | 1KEV | A177P | 0.50 | A | Yes |
| 11 | Alcohol dehydrogenase^a^ | 1KEV | H222P | -6.50 | A | Yes |
| 12 | Alcohol dehydrogenase^a^ | 1KEV | L275P | -2.30 | A | Yes |
| 13 | Alcohol dehydrogenase^a^ | 1KEV | L316P | 10.80 | A | Yes |
| 14 | Alcohol dehydrogenase^a^ | 1KEV | A347P | 2.60 | A | Yes |
| 15 | Chitinase^a^ | 1KFW | G93P | 1.20 | A | Yes |
| 16 | Chitinase^a^ | 1KFW | G254P | -0.65 | A | No |
| 17 | Cel12A^a^ | 1OLR | C206P | -9.20 | A | No |
| 18 | MBP^b^ | 1OMP | K34P | 2.20 | A | Yes |
| 19 | MBP^b^ | 1OMP | D41P | 0.30 | A | Yes |
| 20 | MBP^b^ | 1OMP | E78P | -0.90 | A | Yes |
| 21 | MBP^b^ | 1OMP | D82P | 0.00 | A | Yes |
| 22 | MBP^b^ | 1OMP | D287P | 0.70 | A | Yes |
| 23 | MBP^b^ | 1OMP | V357P | 0.60 | A | Yes |
| 24 | Protein G^c^ | 1PGA | T2P | -6.60 | A | Yes |
| 25 | Protein G^c^ | 1PGA | G9P | -17.00 | I | Yes |
| 26 | Protein G^c^ | 1PGA | K10P | -8.40 | A | Yes |
| 27 | Protein G^c^ | 1PGA | V21P | 6.20 | A | Yes |
| 28 | Protein G^c^ | 1PGA | A23P | -1.40 | A | Yes |
| 29 | Protein G^c^ | 1PGA | A24P | -4.60 | A | Yes |
| 30 | Protein G^c^ | 1PGA | T25P | -20.90 | I | No |
| 31 | Protein G^c^ | 1PGA | V29P | -22.60 | I | Yes |
| No | Protein | PDB id | Mutation | ΔT_m_/ΔΔG^#^ | Phenotype^*^ | Correct prediction |
| 32 | Protein G^c^ | 1PGA | D36P | -21.00 | I | No |
| 33 | Protein G^c^ | 1PGA | A48P | -6.80 | A | Yes |
| 34 | Protein G^a^ | 1PGA | A26P | -32.00 | I | Yes |
| 35 | Rop^a^ | 1ROP | D30P | -9.80 | A | No |
| 36 | α-parvalbumin^a^ | 1RTP | A21P | -8.50 | A | Yes |
| 37 | α-parvalbumin^a^ | 1RTP | H26P | 5.60 | A | Yes |
| 38 | Fyn tyrosine kinase^a^ | 1SHF | E24P | -15.90 | I | Yes |
| 39 | Ubiquitin^a^ | 1UBQ | E34P | -30.30 | I | Yes |
| 40 | RBP^b^ | 1URP | K270P | -2.30 | A | Yes |
| 41 | Tryptophan synthase α-subunit^a^ | 1WQ5 | S6P | -5.20 | A | Yes |
| 42 | Tryptophan synthase α-subunit^a^ | 1WQ5 | E49P | 0.80 | A | No |
| 43 | Fibroblast growth factor-1^a^ | 2AFG | L44P | 3.00 | A | No |
| 44 | LIVBP^b^ | 2LIV | D2P | -0.40 | A | Yes |
| 45 | LIVBP^b^ | 2LIV | K43P | 1.00 | A | Yes |
| 46 | Ribonuclease HI^a^ | 2RN2 | A52P | -5.40 | A | Yes |
| 47 | Ribonuclease HI^a^ | 2RN2 | H62P | 4.10 | A | Yes |
| 48 | Ribonuclease HI^a^ | 2RN2 | Q113P | -2.10 | A | Yes |
| 49 | Thioredoxin^b^ | 2TRX | D2P | 0.70 | A | Yes |
| 50 | Thioredoxin^b^ | 2TRX | V86P | -2.90 | A | Yes |
| 51 | Thioredoxin^b^ | 2TRX | V91P | -4.70 | A | No |
| 52 | Thioredoxin^b^ | 2TRX | A108P | 0.50 | A | No |

^#^ Experimental values ΔT_m_ or ΔΔG, where ΔT_m_ = T_m_(mut) - T_m_(wt), and ΔΔG = ΔG(mut) - ΔG(wt). ΔΔG values are shown in red

^*^ Pro substitution phenotype from experimental data. If ΔT_m_ < -10 °C or if ΔΔG < -0.5 kcal/mol, the phenotype is assumed to be inactive.

^a^ 29 Proline mutants from ProTherm database

^b^ Reference [1]

^c^ Reference [2]

#### References

1. Prajapati RS, Das M, Sreeramulu S, Sirajuddin M, Srinivasan S, et al. (2007) Thermodynamic effects of proline introduction on protein stability. Proteins 66: 480-491.

2. Choi EJ, Mayo SL (2006) Generation and analysis of proline mutants in protein G. Protein Eng Des Sel 19: 285-289.
